# Protocol of a Randomized Controlled Trial of Culturally Sensitive Interventions to Improve African Americans' and Non-African Americans' Early, Shared, and Informed Consideration of Live Kidney Transplantation: The talking about Live Kidney Donation (TALK) study

**DOI:** 10.1186/1471-2369-12-34

**Published:** 2011-07-08

**Authors:** L Ebony Boulware, Felicia Hill-Briggs, Edward S Kraus, J Keith Melancon, Raquel McGuire, Bobbie Bonhage, Mikiko Senga, Patti Ephraim, Kira E Evans, Brenda Falcone, Misty U Troll, Nicole Depasquale, Neil R Powe

**Affiliations:** 1 Division of General Internal Medicine, Department of Medicine, Johns Hopkins School of Medicine, 2024 E. Monument Street, Baltimore, MD 21205, USA; 2 Department of Epidemiology, Johns Hopkins Bloomberg School of Public Health, 615 N. Wolfe Street, Baltimore, Maryland 21205, USA; 3 Welch Center for Prevention, Epidemiology and Clinical Research, Johns Hopkins Medical Institutions, 2024 E. Monument Street, Baltimore, Maryland, 21205, USA; 4 Department of Health Behavior and Society, Johns Hopkins Bloomberg School of Public Health, 615 N. Wolfe Street, Baltimore, Maryland 21205, USA; 5 Division of Nephrology, Department of Medicine, Johns Hopkins School of Medicine, 1830 E. Monument Street, Baltimore, Maryland, 21287, USA; 6 Division of Transplant Surgery, Department of Surgery, Georgetown University Hospital, 3800 Reservoir Road, NW | Washington DC, 20007, USA; 7 National Kidney Foundation of Maryland, 1107 Kenilworth Drive, Baltimore, Maryland, 21204, USA; 8 University of California San Francisco and San Francisco General Hospital, 1001 Potrero Avenue, San Francisco, California 94110, USA

## Abstract

**Background:**

Live kidney transplantation (LKT) is underutilized, particularly among ethnic/racial minorities. The effectiveness of culturally sensitive educational and behavioral interventions to encourage patients' early, shared (with family and health care providers) and informed consideration of LKT and ameliorate disparities in consideration of LKT is unknown.

**Methods/Design:**

We report the protocol of the Talking About Live Kidney Donation (TALK) Study, a two-phase study utilizing qualitative and quantitative research methods to design and test culturally sensitive interventions to improve patients' shared and informed consideration of LKT. Study Phase 1 involved the evidence-based development of culturally sensitive written and audiovisual educational materials as well as a social worker intervention to encourage patients' engagement in shared and informed consideration of LKT. In Study Phase 2, we are currently conducting a randomized controlled trial in which participants with progressing chronic kidney disease receive: 1) usual care by their nephrologists, 2) usual care plus the educational materials, or 3) usual care plus the educational materials and the social worker intervention. The primary outcome of the randomized controlled trial will include patients' self-reported rates of consideration of LKT (including family discussions of LKT, patient-physician discussions of LKT, and identification of an LKT donor). We will also assess differences in rates of consideration of LKT among African Americans and non-African Americans.

**Discussion:**

The TALK Study rigorously developed and is currently testing the effectiveness of culturally sensitive interventions to improve patients' and families' consideration of LKT. Results from TALK will provide needed evidence on ways to enhance consideration of this optimal treatment for patients with end stage renal disease.

**Trial Registration:**

ClinicalTrials.gov number, NCT00932334

## Background

Living related kidney transplantation (LKT) represents an optimal strategy for the treatment of patients with end stage renal disease (ESRD), offering recipients substantially improved length and quality of life when compared to patients receiving dialysis [1]. However, evidence suggests LKT has been consistently underutilized, particularly among ethnic/racial minorities, who have been demonstrated to be up to 50% less likely than non-minorities to receive LKT [2]. Underutilization of LKT has been attributed, in part, to potential recipients' lack of knowledge about LKT [3,4], denial about the need to consider LKT [5], difficulties approaching and identifying donors [6], potential recipients' fears regarding the health of potential donors [7], mistrust of health care and concerns about surgery [8], and suboptimal discussions about LKT between potential recipients, their families, and health care providers [9].

Little is known about effective strategies for improving patients' and families' early consideration of LKT. Consideration of LKT prior to patients' development of ESRD could provide time for both recipients and potential donors (often family members) to fully consider the risks and benefits of LKT, enhancing the probability that their decisions regarding pursuit of LKT align with their personal values. Early consideration of LKT may be particularly important in facilitating and preparing for pre-emptive (before the start of dialysis) LKT-- which is associated with the most superior clinical outcomes [10]. Despite this, studies show most patients, particularly ethnic/racial minorities, do not consider LKT as a treatment option until after they have initiated hemodialysis, and that family and physician discussions about LKT are often suboptimal even when patients report they desire LKT as a treatment [3,9].

Patients who have not yet progressed to ESRD may have unique barriers to considering LKT, including their lack of awareness of the severity of their kidney disease [11] and low perceived susceptibility to kidney disease progression and ESRD [12]. Evidence suggests patients with progressing kidney disease and their families, particularly ethnic/racial minorities, may also have avoidant coping strategies which hinder their collaborative decision making [5]. Patients may also feel they have inadequate skills to initiate discussions about LKT with their families and health care providers, further limiting opportunities for early consideration of LKT [13]. Studies assessing the effectiveness of culturally sensitive approaches to improve patients' early shared, and informed consideration of LKT are needed.

We describe the protocol for a two phase study in which we 1) developed culturally sensitive educational and behavioral interventions to improve patients' early, shared, and informed consideration of LKT and 2) are currently testing the effectiveness of these interventions to improve patients' consideration of LKT in a randomized controlled trial.

## Methods/Design

### Study Design Summary

The Talking about Live Kidney Donation (TALK) study is a two-phase study to design and test the effectiveness of culturally sensitive behavioral and educational interventions on patients' early, shared, and informed consideration of LKT. In Phase 1, we developed culturally sensitive interventions and we developed and piloted data collection tools. In Phase 2, we are currently conducting a randomized controlled trial to assess the effectiveness of interventions to improve patients' shared and informed consideration of LKT. The Johns Hopkins School of Medicine Institutional Review Board has approved all study procedures.

### Phase 1: Development of Culturally Sensitive Educational and Behavioral Interventions

Interventions were developed on the basis of evidence obtained through (1) a review of published scientific literature informing barriers to patients' pursuit of LKT among minorities and non-minorities as well as a review of lay publications (brochures, pamphlets) produced by patient advocacy organizations and government agencies to educate patients on LKT [14-16], and (2) primary data collection from qualitative group interviews of African American and non-African American patients and families with varying levels of experience regarding pursuit of LKT. Full details regarding the conduct of group interviews are published elsewhere [13].

#### Development of Educational Booklet and Video

The educational booklet and video were developed by a team of experts, including a transplant nephrologist, a transplant surgeon, a transplant social worker, a general internist, a behavioral psychologist, experts in developing interventions to address health disparities, experts in creating health literacy sensitive educational materials, and patient advocates from the National Kidney Foundation of Maryland. Educational materials provide complimentary information to encourage patient and families' shared and informed consideration of LKT, including: 1) information about eligibility for LKT, 2) the clinical evaluation required for LKT, 3) the donor selection process, 4) surgical procedures for transplantation and donation, 5), and concerns brought forth about these factors (e.g. safety of procedures, concerns about recovery) identified through the structured group interviews [13].

Both the booklet and the video are designed to be reviewed by patients and their families. The booklet is designed to be read at a fourth to sixth grade reading level and to be sensitive to persons with limited health literacy [17]. It also provides example 'model conversations' which patients and/or family members might use to initiate LKT discussions as well as strategies for handling difficult issues (e.g., concerns about pressuring donors during discussions about LKT (for patients) or methods for approaching potential recipients in need of LKT (for family members)), a glossary of commonly used medical terms related to LKT as well as information about publicly available resources for obtaining further information about LKT. The video features testimonials from African American and non-African Americans patients and families about their experiences with considering LKT (including deliberations about donation and the impact of LKT on their families) and also features physicians and social workers describing factors (e.g. such as the need for social support and regular medical follow up) that patients and families should consider with regard to LKT. The video also addresses African Americans' potential mistrust of the transplant process. The video and booklet were both screened by African American and non-African American patients and families prior to final production and their input was incorporated into the final interventions.

#### Development of behavioral social worker intervention and intervention protocol

We have developed a family-based social worker intervention founded on well-established Social Construction-based Family Problem Solving Theory, [18-20] to address barriers to patients consideration of LKT. The intervention is based on five key principles derived from Family Problem Solving Theory which center on the value of the health care practitioner (social worker) to facilitate the resolution of difficult issues or decisions by encouragement of mutual family discourse and by assisting families' develop strategies for problem management. (Table 1) Additional core principles underlying the intervention include the discussion of all forms of renal replacement therapy as reasonable treatment options and the avoidance of coercion of potential living donors toward donation in all discussions.

**Table 1:** Underlying principles of social worker brief counseling sessions for patients and family members

Core Tenets Underlying Conduct of Family Problem Solving Intervention (General)
• The act of participating in mutual discourse with others enables individuals to create meaning and purpose from their life experiences.
• Problem-solving conversations allow individuals to be involved in a mutual search for and creation of new solutions to their problems.
• A client system is composed of those who are joined for the purpose of dialogue around problems or issues of mutual concern. The clinician must enter into dialogue with the family to become part of the collective view needed to assist in creating new solutions with the family.
• The ways in which family members communicate with one another define their collective view about a problem.
• The role of the clinician is to create space for and facilitate conversations through which the family can find new meanings and mutually acceptable solutions for existing health problems.

**Principles Specific to Family Problem Solving for LKT**

• Discussion and education regarding all treatment options available to patients (including dialysis, deceased kidney transplantation, living related kidney transplantation, and living unrelated kidney transplantation) will occur.
• Encouragement of truly shared decision-making and avoidance of coercion of family members toward donation is a core component of discussions.

During the intervention, a social worker engages potential LKT recipients in one-on-one meetings and, if potential recipients desire, also engages potential recipients and their families in a follow-up family meeting. In one-on-one meetings with potential recipients, the social worker probes to assess specific factors potential recipients perceive inhibit them from progressing through behaviors necessary to achieve LKT (including perceived lack of knowledge regarding LKT, fears regarding the procedure and life after LKT, concerns regarding the health and well-being of potential family donors, and resources necessary for shared decision-making regarding patient's kidney failure). In follow-up meetings with families, the social worker probes to assess specific concerns or barriers family members perceive might inhibit them from pursuing LKT (including strained relationships, financial concerns, and logistical issues). The social worker also probes to assess the extent to which previous family discussions about patients' CKD have occurred, the results of those discussions, whether families have discussed living LKT with patients' physicians, and barriers families perceive for an open discussion about living kidney donation/transplantation with patients' physicians. The social worker's assessments of factors primarily affecting potential recipients' decision-making at the time of the intervention will guide their development of individualized culturally sensitive intervention plans based on the perceived needs of the potential recipient. Thus, goals of patient and family meetings will be based on which factors are felt to be most prominent for each individual potential recipient and their family. The social worker is also available to patients and families for the provision of information regarding LKT as well as for limited facilitation of further family communication. Social workers employed in the intervention have been trained in techniques for brief individual/family therapy by a behavioral psychologist with experience with problem solving interventions. Social workers have also been trained in effective cross-cultural communication and negotiation utilizing the model for education in cultural competence developed by the American Association of Medical Colleges [21].

### Phase 2: Conduct of Randomized Controlled Trial

We are currently conducting a randomized controlled trial to assess the incremental effectiveness of educational materials and the social worker intervention on improving patients' and families' engagement in shared and informed consideration of LKT.

#### Target Population and Eligibility Criteria

We are targeting patients with advanced, progressive chronic kidney disease who have not yet initiated dialysis therapy and their family members for participation. Patients are recruited from five academically affiliated and community-based nephrology practices in the Baltimore, MD metropolitan region, which we have selected to provide an ethnically and socioeconomically diverse population of participants. To identify potential participants with kidney disease, we ask nephrologists from participating practices to identify from their patients with National Kidney Foundation Kidney Disease Outcomes Quality Initiative (KDOQI) Stages 4 or 5 (not dialysis dependent) CKD (glomerular filtration rates ≤ 30 ml/min/1.73 m^2^), those whom the physicians believe are potentially eligible for kidney transplantation based on the following criteria: age 18 to 70; no evidence of cancer within 2 years prior to recruitment date; no evidence of stage IV congestive heart failure; no evidence of end-stage liver disease; no evidence of unstable coronary artery disease; no evidence of pulmonary hypertension; no evidence of severe peripheral vascular disease; no history of HIV; no chronic (debilitating) infections; and no prior kidney transplant.

#### Recruitment of patient participants

We recruit patient participants via both active and passive strategies. We recruit participants passively via brochures and recruitment flyers (encouraging potential participants' self referral) placed in practice waiting rooms and examination rooms. The brochures provide a description of the study goals, participant eligibility criteria, and the contact phone number and e-mail for the coordinating center. We actively identify potentially eligible participants by screening nephrology practices' administrative (i.e. billing codes, Table 2) and clinical records (i.e. using a modified estimation equation derived from the Modification of Diet in Renal Disease Study [22], which incorporates serum creatinine, patient, age, race, and gender to estimate glomerular filtration rate) on all patients seen at the clinical practice sites over the previous 12 months to identify persons with CKD stages 4 or 5 (non-dialysis dependent). We have obtained a HIPAA waiver to screen medical records to confirm potential eligibility among potential participants identified through billing codes. Once we have identified potentially eligible participants through screening, we forward their names to their nephrologists for confirmation of their potential eligibility. We mail all potentially eligible participants a letter from their physician group advising them that they are eligible to participate in the study along with a letter from the study principal investigator with a brief summary of the study eligibility criteria, objectives, what they would be asked to do as a study participant, and information on how to contact the study recruiters by telephone. Letters include self-addressed stamped envelopes with standard refusal postcards allowing potential participants to refuse participation. We provide potentially eligible participants fourteen (14) days to refuse either by mail or phone. We do not contact potentially eligible persons who refuse. If the study coordinating center does not hear from the participant, trained recruiters attempt to contact potential participants by telephone and assess their willingness to participate in the study using a standardized telephone script and oral consent process. Upon confirmation of their eligibility to participate, we randomly assign participants into intervention groups.

**Table 2:** Administrative codes used to identify potential participants with stages 4 and 5(non-dialysis dependent) CKD for the TALK study

Condition	ICD-9 Code
Diabetic nephropathy	250.4, 250.40, 250.41, 250.42, 250.43

Hypertensive nephropathy	403.00-403.91

Hypertensive heart and kidney disease	404.00-404.93

Nephrotic syndrome	581

Chronic kidney disease	585, 585.4, 585.5, or 585.9

Disorders resulting from impaired renal function	588

#### Recruitment of family member participants

At the time of their one (1) month follow up telephone assessment, we ask all patient participants (regardless of intervention assignment) to identify a family member they feel they would involve in an important medical consideration, such as the consideration to pursue LKT to participate in a telephone questionnaire. We offer patient participants two options for contacting family members: 1) patient participants may contact family members or friends themselves and invite them to contact the study coordinating center, or 2) patient participants may let family members or friends know that they have provided their family members' contact information to the study and that the coordinating center will contact the family member. We recruit family members using a prepared telephone script and oral consent process.

For patient participants randomized to receive the social worker intervention (see intervention groups below), we ask participants to invite family members to a follow-up visit with the social worker. Prior to attending the follow up social worker session, we contact family members via telephone to obtain their consent for participating in the study using a prepared telephone script and oral consent process. Some participants bring additional family members, friends, or other non-relatives to visits beyond those consented. We therefore also obtain written consent from family members or friends attending social worker meetings.

#### Randomization

Using blind and secure allocation by computer, we randomly assign participants to one of three intervention arms: 1) control group ("Usual Care"); 2) video and booklet intervention only ("TALK Program"); and 3) video, booklet plus social worker intervention ("TALK Plus Program"). Randomization is blocked by recruitment site to ensure equal allocation within each site.

#### Usual Care Group

Participants randomly assigned to receive Usual Care proceed with their usual clinical care with their nephrologists as already routinely implemented by their physicians. Since all patient participants have stage 4 CKD at the time of recruitment, many have already had some discussions of options for renal replacement therapy with their physicians (including LKT). Many practices from which patient participants are recruited routinely refer patients to "pre-dialysis education" group sessions led by dialysis social workers and nurses to discuss the process of preparation for dialysis and transplantation.

#### Intervention Groups

Patient participants randomly assigned to receive the TALK Program receive the culturally sensitive educational booklet and video at the time of enrollment. We provide participants the option of reviewing these materials on their own or with study staff in their homes or at a public location of their choice. Study staff members also encourage patient participants to share the materials with their family members or friends. Participants randomly assigned to receive the TALK-Plus Program receive the culturally sensitive educational booklet and video at the time of enrollment in a similar fashion with a similar approach to the TALK Plus assignment. In addition, participants in this group are invited to participate in an initial 60-minute counseling session with a licensed social worker experienced in working with patients and families undergoing evaluation for transplant surgery followed by a second 60-minute session with the social worker and their families, if patient participants desire. All participant meetings are audio-recorded and later transcribed verbatim, excluding names of participants for analysis of participant meeting content.

#### Data collection, follow-up and outcomes

##### Measures Assessed Through Structured Interviews

All patient participants enrolled in the study are assessed using structured interviews administered by telephone at baseline, 1 month, 3 months, and 6 months following recruitment and enrollment in the study.

**Figure 1:**
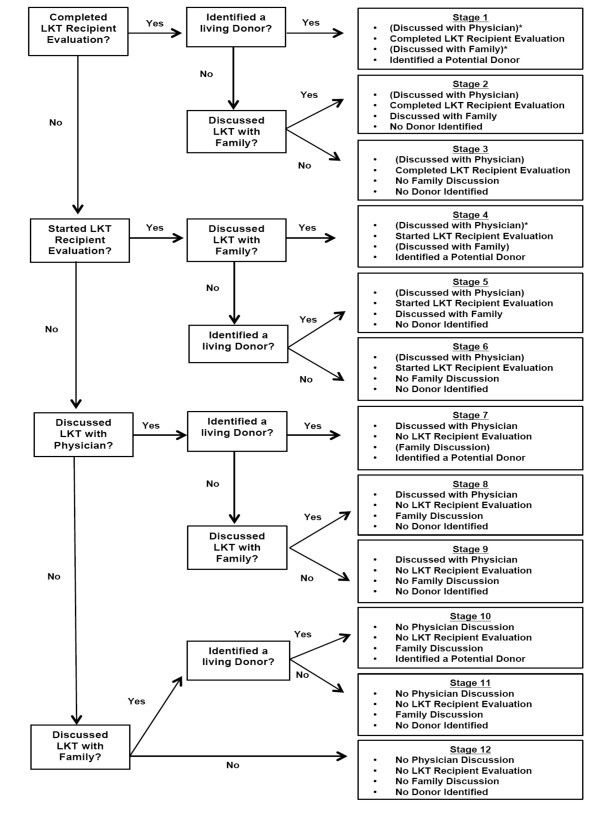
**Schematic describing determination of key behaviors representing consideration of LKT in the TALK study**. *Participants assumed to have discussed LKT with physicians if they had begun their clinical evaluation for LKT; Participants also assumed to have discussed LKT with their families if they identified a donor.

The primary outcome is change in participants' consideration of LKT over time. This outcome is measured as participants' movement through 12 possible behavioral 'stages' reflecting their completion of key steps toward considering LKT, including (1) preparation for and/or execution of patient-family discussions about LKT; (2) preparation for and/or execution of patient-physician discussions about LKT; (3) preparation for and/or completion of evaluation for LKT; (4) completion of the LKT recipient evaluation, and (5) identification of a potential live kidney donor. To assess these behaviors, we ask participants a series of questions to which they can answer 'yes' or 'no' (e.g. "Have you already completed the testing process to get a kidney transplant?"). We categorize participants into one of the 12 stages based on their answers. Once we have categorized the participants, we also assess potential barriers to consideration of LKT, including the barriers to discussions with family and providers about LKT (e.g. difficulty discussing LKT with physician, competing priorities, uncertainty about desire for LKT). (Figure 1)

Other measures include assessment of participants' (1) access to and sources of information about LKT; (2) barriers to obtaining information on LKT; (3) prior discussions with health care providers about LKT; (4) perceived satisfaction and patient-centeredness of their relationship with their nephrologist; (5) preferred role in decision-making about LKT; (6) beliefs about the benefits of LKT; (7) knowledge and interest regarding LKT; (8) concerns about the risks of LKT to themselves and to donors; (9) family structure and relationships; (10) depression and social support; (11) religiosity/spirituality; (12) financial stress, (13) health literacy, and (14) sociodemographic characteristics. (Table 3) For patient participants assigned to the TALK and TALK-Plus groups, we also assess their perceptions of the helpfulness of the video and booklet.

**Table 3:** Schedule of patient and family participant assessments in the TALK study

	PATIENT				FAMILY	
**Measure**	**Baseline**	**1** **Month**	**3** **Month**	**6** **Month**	**Baseline**	**3** **Month**

**Exposure to information about Kidney Disease Treatment**						

Dialysis Information	X	X	X	X	X	X

Kidney Transplant Information	X	X	X	X	X	X

Live Donor Kidney Transplant Information	X	X	X	X	X	X

**Discussions with Health Care Providers about Kidney Disease Treatment**						

Prior Discussions with Social Worker(s)	X				X	

Occurrence of Physician Discussions-Primary Care, Nephrologists	X					

Perception of Centeredness in Patient-Physician Discussions [34]	X		X	X		

Satisfaction with Patient-Physician Discussions	X					

Physician Recommendations regarding LKT	X					

**Belief & Knowledge about Treatment for Kidney Failure, Interest in LKT**						

Beliefs About Treatment for Kidney Failure	X	X	X	X		

Knowledge of LKT	X	X	X	X	X	X

Interest in LKT	X	X	X	X		

Consideration of LKT (stage placement)	X	X	X	X		

**Pursuit of LKT, Barriers to Completing Behavior Stages**						

Quality of Family Discussion	X	X	X	X	X	X

Information on Donor	X	X	X	X		

Barriers to Patient-Family Discussion	X	X	X	X		

Barriers to Patient-Physician Discussion	X	X	X	X		

Barriers to Starting Evaluation	X	X	X	X		

Barriers to Completing Evaluation	X	X	X	X		

**Mediators and Correlates of Pursuit of LKT**						

Trust in Medical Care [35-37]	X					

Patient Activation Measure-Short Form [38]	X	X	X	X		

Decision Self-Efficacy [39]	X	X	X	X		

Family Structure Inventory	X					

Family Assessment Device (General, Communication, and Problem Solving Scales)[40,41]	X		X	X	X	X

Depressed Mood-Prime MD/PHQ [42]	X		X	X		

Medical Outcomes Study (MOS) Social Support Scale [43]	X		X	X		

Spirituality & Religion [44]	X					

Trait Hope Scale [45]	X	X	X	X		

Personal Financial Well-being Scale [46]	X					

Sociodemographic Information	X				X	

**Assessment of Booklet and Video**						

Assessment of Booklet and Video		X	X	X	X	X

**Health Literacy**						

Rapid Estimate of Adult Literacy in Medicine (REALM) [47]	X					

Patient participants' family members are assessed once after patient participants' 1-month assessments and once after patient participants' 3-month assessment via structured interviews administered via telephone. Assessments include family members': (1) access to and sources of information regarding LKT (including whether family members have reviewed intervention materials with patient participants); (2) knowledge about LKT; (3) presence and quality of family discussions about LKT.

#### Social Worker Assessments

For participants randomized to receive social worker meetings (TALK Plus Program) social workers document potential barriers to LKT elucidated by patients and families, and assess immediate outcomes of patient/family discussions, including: (1) whether patients and families arrived at consensus regarding mutual patient/family solutions, (2) presence (or lack thereof) of plans agreed upon by patients and families to discuss LKT with patients' physicians, and (3) presence (or lack thereof) of plans agreed upon by patients and families to pursue evaluation for LKT.

### Statistical Considerations

#### Data Analysis

Randomly assigned intervention group (i.e. Usual Care, Talk Study, TALK Plus) will be the main independent variable for intent-to-treat analysis [23]. The co-primary outcomes are change in consideration of LKT at 3 and 6 months. To evaluate the effectiveness of the intervention on outcomes, we will utilize generalized estimating equations, which account for the longitudinal nature of the trial and incorporate baseline, 3 and 6 months measurements as well as study site characteristics and other baseline characteristics found not to be balanced by randomization. Secondary analyses will include exploratory analyses stratifying participants according to ethnicity/race to assess differences intervention effectiveness among African Americans and non-African Americans. We will also perform exploratory analyses among persons within subgroups defined post-randomization--for example, analyses among persons with greater versus less knowledge of LKT at baseline. We will conduct primary analyses under the assumption that data is missing at random, however, we will also perform sensitivity analyses based on other scenarios (i.e. patterns of missing data) to evaluate the robustness of our assumptions.

#### Sample Size and Power

Our main analysis will assess the proportion of participants in each randomized group that has moved one additional step 'forward' in consideration of LKT over study follow up. We are not aware of other studies assessing this outcome among patients and families who have not yet initiated dialysis therapies. We conservatively assume that approximately 30% of our participants, who have not yet initiated dialysis, will consider LKT as a treatment option under usual care at follow up [5,24,25]. A recently performed randomized controlled trial indicated that engagement of family in discussions about LKT improved rates of donor evaluations up to 2-fold [26]. Based on this data, we assume that our educational interventions (video and booklet) could improve consideration by approximately 2-fold and that the behavioral intervention (social worker) would additionally improve the effectiveness of the intervention by 10% (i.e. 30%, 60%, 70% consideration of LKT at follow up in the usual care, Talk, and Talk Plus groups, respectively). Under these assumptions, for two-sided 0.05 level test of the null hypothesis, recruitment of 40 participants per group should provide approximately 95% power to detect statistically significant differences in consideration of LKT.

## Discussion

Despite the success of LKT as a treatment strategy for ESRD, improvements in utilization have been largely stagnant in recent years, and ethnic/racial disparities in utilization of LKT persist [2,27]. Although efforts to improve early consideration of LKT are advocated [28], effective strategies for engaging ethnic/racial minority and non-minority patients and their families in early, shared and informed consideration of LKT have not been studied. The TALK study has rigorously developed and is currently testing the effectiveness of novel strategies to improve ethnic/racial minority and non-minority patients' and families' early, shared and informed consideration of LKT as treatment option.

The TALK study represents one of the first efforts to explicitly design culturally sensitive interventions to improve African American' and non-African Americans' early consideration of LKT. Prior studies of educational interventions among patients with progressive chronic kidney disease have not assessed the effect of early education on patients' or their families' consideration of LKT and have not examined whether interventions narrow ethnic/race differences in consideration of treatments among United States patients [29,30]. A recent study among patients who were already treated on dialysis demonstrated home visits performed by health professionals to engage patients and families in shared and informed decision-making about LKT were effective in improving minority and non-minorities' consideration of LKT [26]. However, this study did not address the effectiveness of earlier educational interventions to improve patients' and their families' consideration of LKT. Interventions supporting patients' and families' earlier consideration of LKT could provide patients and families needed time to discuss LKT and initiate steps toward pursuit before patients and families experience commonly reported psychosocial and physical stress associated with dialysis initiation [31-33].

Our efforts to identify both shared and differing concerns about pursuing LKT among minority and non-minority patients and families represent a novel strategy for developing culturally sensitive interventions to improve consideration of LKT and could enhance interventions' effectiveness. If effective, interventions could serve as a model for future programs seeking to provide resources to support ethnic/racial minority and non-minority patients' and families' early and informed decisions about LKT. Our collection of information on factors contributing to interventions' success (e.g. the patient-physician relationship and family members' perspectives on the process of considering LKT in discussions) will provide additional valuable insight into the mechanisms through which interventions may act as well as ways in which interventions might be further tailored to optimize their effectiveness.

In summary, the TALK study has rigorously developed and is currently testing the effectiveness of culturally sensitive educational and behavioral interventions to improve patients' and families' early and informed consideration of LKT. Results from TALK will provide needed evidence regarding ways to enhance consideration of this optimal treatment strategy among African American and non-African American patients and families.

## Abbreviations

LKT: Live Kidney Transplantation; ESRD: End-Stage Renal Disease; TALK: Talking About Living Kidney Donation.

## Competing interests

The authors declare that they have no competing interests.

## Authors' contributions

LEB conceived, designed, and conducted the study and drafted the manuscript. FHB contributed to the conception, design, and conduct of the study and the drafting of the manuscript. EK contributed to the conception, design, and conduct of the study and the drafting of the manuscript. JKM contributed to the conception, design, and conduct of the study and the drafting of the manuscript. RM contributed to the conception, design, and conduct of the study. BB contributed to the conception, design, and conduct of the study. MS contributed to the conception, design, and conduct of the study and the drafting of the manuscript. KEE contributed to the conception, design, and conduct of the study and the drafting of the manuscript. PE contributed to the conception, design, and conduct of the study and the drafting of the manuscript. MU contributed to the conception, design, and conduct of the study and the drafting of the manuscript. ND contributed to the drafting of the manuscript. NRP contributed to the conception, design, and conduct of the study and the drafting of the manuscript. All authors approved the final manuscript.

## Pre-publication history

The pre-publication history for this paper can be accessed here:

http://www.biomedcentral.com/1471-2369/12/34/prepub
